# The Association Between Physical Activity, Sedentary Time, and Sleep with Risk of Hepatocellular Carcinoma in Patients with Cirrhosis

**DOI:** 10.1007/s10620-025-09527-5

**Published:** 2025-11-07

**Authors:** Samuel Ma, Yossef Alsabawi, Fasiha Kanwal, Hao Duong, Laith Elserag, Amit G. Singal, Nicole E. Rich, Saira Khaderi, Sumeet K. Asrani, Hashem B. El-Serag, Aaron P. Thrift

**Affiliations:** 1 School of Medicine, Baylor College of Medicine, Houston, TX USA; 2 School of Medicine, University of Texas Rio Grande Valley, Edinburg, TX USA; 3 Section of Gastroenterology and Hepatology, Department of Medicine, Baylor College of Medicine, Houston, TX USA; 4 Veterans Affairs Health Services Research and Development Service Center for Innovations in Quality, Effectiveness, and Safety (IQuESt), Michael E. DeBakey Veterans Affairs Medical Center, Houston, TX USA; 5 University of Texas at Austin, Austin, TX USA; 6 Division of Digestive and Liver Diseases, Department of Medicine, UT Southwestern Medical Center, Dallas, TX USA; 7 Baylor University Medical Center, Dallas, TX USA; 8 Section of Epidemiology and Population Sciences, Department of Medicine, Baylor College of Medicine, Houston, TX USA; 9 Dan L Duncan Comprehensive Cancer Center, Baylor College of Medicine, One Baylor Plaza, MS: BCM307, Room 621D, Houston, TX 77030 USA

**Keywords:** Liver cancer, Prevention, Lifestyle, Epidemiology

## Abstract

**Background:**

The majority of hepatocellular carcinoma (HCC) cases arise in patients with cirrhosis, with known major risk factors of hepatitis B (HBV) or C (HCV) infection, metabolic dysfunction-associated steatotic liver disease (MASLD), and alcohol-associated liver disease (ALD). However, the impact of additional risk factors that act independently or jointly on the pathogenesis of HCC in cirrhosis patients is unknown. The aim of this study was to determine whether sleep, sedentariness, or physical activity levels play a role in the progression of cirrhosis to HCC.

**Methods:**

We systematically collected data on sleep, sedentary time, and physical activity from the ongoing prospective study, the Texas HCC Consortium Cohort, which recruited patients with cirrhosis from December 2016 and followed up until HCC development, death, or May 2024. Hazard ratios (HRs) were calculated using Fine-Gray competing risk regression models to evaluate the independent association between sleep, sedentary, and physical activity levels with the risk of HCC overall and in groups stratified by major HCC risk factors.

**Results:**

Of 3940 patients with cirrhosis (mean age = 59.9 years, 39.1% women), 9.3% of patients had active HCV, 34.6% had MASLD, 41.5% had MetALD, and 3.2% had ALD only. In total, 208 patients developed incident HCC (annual incidence rate, 2.37% [95% CI 2.07, 2.72%]). After adjustment for demographic and clinical factors, absent levels of light physical activity (HR = 0.92 [0.57–1.47]), excessive sitting time (HR = 0.91 [0.57–1.44]), and reduced sleep duration (HR = 0.92 [0.65–1.29]) were not associated with an increased risk of HCC development. There were no associations with increased risk of HCC development across all sub-groups examined.

**Conclusions:**

In this study, the risk of HCC in patients with cirrhosis was not significantly affected by level of light physical activity, sedentariness, or sleep across all major etiologies of cirrhosis.

**Supplementary Information:**

The online version contains supplementary material available at 10.1007/s10620-025-09527-5.

## Introduction

Globally, liver cancer is the sixth most common cancer and the third leading cause of cancer-related mortality [1]. Hepatocellular carcinoma (HCC) is the most common primary cancer of the liver. Although the incidence rates of HCC in the United States peaked between 2009 and 2015 after a sharp four-decade increase [2, 3], HCC still carries a significant healthcare and patient burden for the U.S. population [4–6].

Most cases (85%-90%) of HCC arise in patients with cirrhosis, and HCC is the leading cause of death in patients with compensated cirrhosis [7, 8]. Major etiologic risk factors for cirrhosis and HCC include hepatitis C virus (HCV) and hepatitis B virus (HBV) infections, metabolic dysfunction-associated steatotic liver disease (MASLD; formerly called non-alcoholic fatty liver disease), and alcohol-associated liver disease (ALD) [9, 10]. In recent years, the etiology of cirrhosis has gradually shifted from active HBV and HCV to MASLD and ALD [11, 12].

Many patients with cirrhosis do not develop HCC, and among those who do, the progression to HCC occurs at different rates [11]. The characterization of the modifiable factors that influence the occurrence and speed of progression to HCC is an ongoing area of research [13–16]. Insufficient duration and quality of sleep, excessive sitting time, and low levels of physical activity are potentially modifiable risk factors that are associated with a well-established increase in all-cause mortality, cardiovascular disease, and total cancer risks [17–20]. Furthermore, these specific modifiable risk factors that influence metabolic health were found to also increase the risk of MASLD development, suggesting that poor physical condition and poor metabolic health play a significant and modifiable role [21, 22]. Previous studies of HCC have shown that variations in physical activity levels, sitting time, and sleep patterns may be associated with HCC risk [17, 18, 23]. However, whether these factors also confer HCC risk among patients with cirrhosis is unknown.

In this prospective cohort study, we assessed the potential independent and joint effects of sleep duration, sedentariness, and light physical activity levels on the risk of cirrhosis progression to HCC both overall and across different major HCC etiological risk factors. We hypothesized that increased sleep duration and light physical activity levels, and reduced sedentariness, would be associated with lower risk of HCC. This is the first study investigating this relationship and its findings could be used to inform prevention efforts such that interventions may help reduce the burden of HCC among patients with cirrhosis.

## Methods

### Study Population

We used data from patients recruited into the Texas HCC Consortium Cohort (THCCC). This study was approved by the Institutional Review Board at all recruitment sites. All participants provided written informed consent to participate.

The THCCC is an ongoing prospective cohort study that recruits patients with cirrhosis from nine institutions in four cities in Texas, the details of which have been previously reported [24]. For the current analysis, we included THCCC participants enrolled between December 2016 and May 17, 2024, with follow-up until May 20, 2024. Cirrhosis diagnosis was based on predefined criteria for liver histology, radiology, liver elastography, or serum biomarkers. We excluded patients with uncontrolled hepatic decompensation, past or present HCC, or presence of non-hepatic cancer at baseline [11]. At the time of consent, patients self-completed a study survey and research assistants abstracted data from the patients’ electronic medical records (EMRs), including (1) clinician-recorded diagnoses, including cirrhosis etiology (e.g., HCV, HBV, ALD, MASLD), complications of liver disease (ascites, varices, encephalopathy), and liver-related treatments; (2) liver imaging and liver biopsy results; and (3) laboratory data (± 12 months from date of consent). Patients were scheduled for 6-monthly visits as part of routine clinical care and followed until HCC diagnosis, liver transplantation, or death. They received liver ultrasound, CT, or MRI for HCC surveillance per the treating physician's decision [25].

## Outcome

Our primary outcome was the development of incident HCC at any time after enrollment. HCC was defined according to the American Association for the Study of Liver Diseases criteria, including histological or radiological diagnosis using characteristic appearance (arterial enhancement and delayed washout) on triple-phase CT or MRI (Liver Imaging Reporting and Data System [LI-RADS] 5) [26]. We also included those with suspicious lesions (LI-RADS 4) that were reviewed in multidisciplinary tumor boards and treated as HCC and adjudicated centrally [27]. EMR reviews were conducted for all participants at 3-month intervals to capture incident HCCs, liver transplantation, and death dates. We also linked the records with the Texas Cancer Registry to identify potential HCC outside of the study follow-up. All study sites have multidisciplinary HCC tumor boards, and, for the current analysis, the date of final confirmation was used as the date of HCC diagnosis [24].

## Measures of Physical Activity, Sedentary Time and Sleep

Patients completed a baseline study survey that ascertained information on light physical activity levels, sitting (sedentary) time, and sleep duration. As such, while possible, any recall bias is not likely to be differential with regards to future HCC state because the data were collected at baseline when everyone in the study did not have HCC. As for physical activity, participants were asked to report the frequency (number of hours/week in pre-defined categories: never/rarely, < 1, 1–3, 4–7, > 7 h/week) dedicated to light physical activities (e.g., walking, bowling, light gardening, fishing) in the past year. Sedentary time was assessed by inquiring about, in the last 7 days, the total hours/day usually spent sitting during work, doing coursework and during leisure-time (sitting at a desk, visiting friends, reading or sitting or lying down to watch television) on a weekday. This data were originally reported as a continuous variable, then broken down into meaningful cutoffs for analysis based on the study population (< 4, 4 −  < 6, 6–8, > 8 h/day). For sleep duration, participants were asked, on average, how many hours of sleep per night they had during the last year (≤ 5, 6, 7, 8, 9, 10, ≥ 11 h).

## Covariables

We recorded patients’ age at study enrollment, sex, and a constructed variable from self-reported race and ethnicity in the survey (non-Hispanic white, non-Hispanic Black, Hispanic, and other groups). Overweight was defined as body mass index (BMI) ≥ 25 kg/m^2^, which was calculated using height and weight values at time of enrolment. We used a survey to ascertain alcohol use that classified alcohol use status as lifetime abstention (never), former, or current use. We defined smoking status as never, past, and current smokers based on self-report using the baseline survey. MASLD was defined as having at least one of the following five conditions: BMI equal to or greater than 25 (23 for Asians), waist circumference (WC) greater than 94 cm for men or 80 cm for women, or type 2 diabetes, hypertension, elevated triglycerides, or elevated cholesterol [25].

- Height, weight, and waist circumference were measured at enrollment.
- Hypertension was defined based on the patient’s self-reported conditions.
- Diabetes and dyslipidemia (elevated cholesterol or triglycerides) were defined based on the patient’s self-reported conditions, prior treatment with diabetes medications, or treatments for dyslipidemia at any time before enrollment.
- ALD was defined as having a clinician-recorded diagnosis of ALD or patients’ self-report of former heavy or any current use of alcohol.
- HCV (active/cured) is defined as having treatment for HCV and/or achieving sustained virologic response (SVR) or having a reported HCV RNA positive result at enrollment. Those who did not achieve SVR at enrollment are considered to have active HCV at that time.
- HBV was defined as having HBsAg positive or HBV DNA positive at enrollment, or having a clinician-recorded diagnosis of HBV, or a patient's self-report of HBV.

We categorized etiologic risk factors based on their relative risk strength, mutually exclusively: active HCV regardless of other conditions; others (HBV, hereditary hemochromatosis, Wilson’s disease, alpha-1 antitrypsin deficiency, or autoimmune hepatitis) regardless of MASLD and ALD; ALD only; MASLD only; and MetALD for those with both ALD and concomitant MASLD [16, 28, 29].

## Statistical Analysis

Patients with cirrhosis were followed from the date of enrollment in THCCC to the development of HCC, death, liver transplantation, or end of follow-up on May 20, 2024. We estimated hazard ratios (HR) and 95% confidence intervals (95% CI) for associations with incident HCC risk using Fine-Gray competing risk regression models, which accounted for the competing risk of death and liver transplant. We examined associations with light physical activity, sedentary time, and sleep duration using multivariable models adjusted for age, gender, race/ethnicity, BMI, and cirrhosis etiology. We also performed these analyses in subgroups defined by cirrhosis etiology and age, diabetes, BMI categories, sex, and alcohol categories to examine for potential differences in the associations with HCC risk. Finally, we examine the joint effects of sleep duration, physical activity levels, and sedentary time on HCC risk by creating a composite variable. For each exposure, we created dichotomous variables and then created a single variable which compared combinations of the three variables with the reference group including individuals with highest level of physical activity (> 7 h weekly), lowest sitting time (< 4 h per day), and more sleep (> 6 h per night), based on the distribution of data in our study population. Analyses were conducted using SAS version 9.4 and R studio.

## Results

We included data from 3940 patients with cirrhosis in the THCCC (Table 1). During a total of 8762.92 person-years of follow-up, 208 patients developed HCC at an annual incidence rate of 2.37% (95% CI 2.07, 2.72%). The mean age of patients at the time of enrollment was 59.9 years (SD = 10.6), 60.9% of the cohort was male, 32.6% Hispanic, 13.5% non-Hispanic Black, 51.2% non-Hispanic White, and 2.6% other race/ethnicity. Most patients were overweight or obese (81.1%), 45.3% had diabetes, 11.7% reported current alcohol drinking, and 17.5% reported current smoking. MetALD was the leading cause of cirrhosis in our patient cohort (41.5%), followed by MASLD (34.6%), active HCV infection (9.3%), and ALD alone (3.2%).

**Table 1:** Select characteristics of cirrhosis patients (*N* = 3,940) in the Texas Hepatocellular Carcinoma Cohort (THCCC)

**Characteristics**		**N (%)**
Age, years	Mean (SD)	59.9 ± 10.6
	< 55	1101 (27.9%)
	55- < 65	1513 (38.4%)
	≥ 65	1326 (33.7%)
Sex		
	Male	2401 (60.9%)
	Female	1539 (39.1%)
Race/Ethnicity		
	Non-Hispanic White	2018 (51.2%)
	Non-Hispanic Black	533 (13.5%)
	Hispanic	1286 (32.6%)
	Other	103 (2.6%)
Alcohol use		
	Never	1194 (30.3%)
	Former	2209 (56.1%)
	Current	462 (11.7%)
	Missing/Unknown	75 (1.9%)
Smoking status		
	Never	1724 (43.8%)
	Former	1391 (35.3%)
	Current	691 (17.5%)
	Missing/Unknown	134 (3.4%)
Body mass index	Mean (SD)	31.1 ± 7.13
Overweight		3194 (81.1%)
Diabetes		1784 (45.3%)
Etiology of cirrhosis		
	Active HCV	367 (9.3%)
	MASLD only	1363 (34.6%)
	ALD only	126 (3.2%)
	MetALD	1633 (41.5%)
	Other*	451 (11.4%)

*ALD* alcohol-related liver disease, *HCV* hepatitis C virus, *MASLD* metabolic dysfunction-associated steatotic liver disease, *MetALD* metabolic and alcohol-related liver disease

^*^Other: HBV hereditary hemochromatosis, Wilson’s disease, alpha-1 antitrypsin deficiency, autoimmune hepatitis or unspecified ALD

When asked about the amount of light physical activity performed weekly, 35.8% of patients had > 7 h per week, while 13.5% reported rarely or never engaging in light physical activity. For sedentary time, 18.9% reported sitting for > 8 h per day, while 20.6% spent < 4 h of their day sitting. Most of the cohort (72.9%) slept 6 or more hours per night.

Table 2 and Tables S1-S3 describe the associations between light physical activity, sedentary time, and sleep duration with risk of progression to HCC, adjusted for age, sex, race/ethnicity, BMI, and cirrhosis etiology. We found no association with physical activity. Compared to cirrhosis patients with > 7 h/week of light physical activity, HCC risk was not different from those who were never or rarely physically active (adjusted HR, 0.92; 95% CI 0.57–1.47). This lack of association was reflected across all levels of light physical activity. Increased sedentary time was also not associated with increased risk of progression to HCC. When compared to cirrhosis patients sitting for < 4 h/day, HCC risk was also no different for cirrhosis patients with sedentary time exceeding 8 h per day (adjusted HR, 0.91; 0.57–1.44). Finally, HCC risk was no different between cirrhosis patients sleeping < 6 h per day compared to those sleeping ≥ 6 h per day (adjusted HR, 0.92; 95% CI 0.65–1.29). When comparing a reference group of study participants with the highest level of physical activity (> 7 h weekly), lowest sitting time (< 4 h per day), and more sleep (> 6 h per night) (*n* = 653; 16.5%), the group with lowest levels of physical activity, the most sitting, and least sleep (*n* = 325; 8.25%) there was still no increased risk of cirrhosis progression to HCC (adjusted HR, 1.18; 95% CI 0.64–2.16). When stratified by cirrhosis etiology (active HCV, MASLD, and MetALD), there were no associations with physical activity level, sedentary time, or sleep duration across all etiologies of cirrhosis (Table 3). We found no associations of physical activity level, sedentary time, or sleep duration with HCC risk in analyses stratified by age group (< 55, 55–65, and 65 +), sex, obesity status, diabetes status, and alcohol use (never, former, and current) (Tables S4-S9).

**Table 2:** Associations of light physical activity, sedentary time, and sleep duration with risk of hepatocellular carcinoma in patients with cirrhosis

Variable	N		Unadjusted HR (95% CI)	Adjusted HR* (95% CI)
Light physical activity				
	530	Never/Rare	1.02 (0.65–1.58)	0.92 (0.57–1.47)
	342	< 1 h weekly	0.86 (0.49–1.49)	0.79 (0.45–1.39)
	821	1–3 h weekly	1.15 (0.80–1.65)	1.13 (0.78–1.65)
	701	4–7 h weekly	0.99 (0.67–1.48)	0.94 (0.62–1.41)
	1409	> 7 h weekly	Reference	Reference
Sedentary time				
	812	< 4 h/day	Reference	Reference
	920	4- < 6 h/day	0.85 (0.56–1.29)	0.88 (0.57–1.35)
	1126	6–8 h/day	1.16 (0.79–1.69)	1.12 (0.75–1.65)
	744	> 8 h/day	1.00 (0.64–1.57)	0.91 (0.57–1.44)
Sleep duration				
	2874	≥ 6 h/day	Reference	Reference
	851	< 6 h/day	0.96 (0.68–1.34)	0.92 (0.65–1.29)

^*^Adjusted for age, sex, race/ethnicity, etiology, and body mass index

**Table 3:** Associations of light physical activity, sedentary time, and sleep duration with risk of hepatocellular carcinoma in patients with cirrhosis, stratified by etiology of cirrhosis

		Active HCV		MASLD Only		MetALD	
Variable		Unadjusted	Adjusted*	Unadjusted	Adjusted*	Unadjusted	Adjusted*
		HR (95% CI)	HR (95% CI)	HR (95% CI)	HR (95% CI)	HR (95% CI)	HR (95% CI)
Light physical activity							
	Never/Rare	0.88 (0.32–2.43)	0.91 (0.30–2.71)	0.96 (0.45–2.05)	0.91 (0.42–1.97)	0.65 (0.30–1.39)	0.59 (0.27–1.29)
	< 1 h weekly	0.79 (0.18–3.52)	0.72 (0.16–3.29)	0.85 (0.35–2.07)	0.78 (0.32–1.94)	0.73 (0.30–1.75)	0.64 (0.26–1.55)
	1–3 h weekly	1.01 (0.36–2.88)	0.88 (0.31–2.50)	1.25 (0.68–2.30)	1.17 (0.62–2.22)	1.01 (0.59–1.74)	0.99 (0.56–1.74)
	4–7 h weekly	1.01 (0.36–2.85)	0.90 (0.33–2.49)	0.86 (0.42–1.74)	0.84 (0.40–1.74)	0.94 (0.52–1.70)	0.91 (0.49–1.67)
	> 7 h weekly	Reference	Reference	Reference	Reference	Reference	Reference
Sedentary time							
	< 4 h/day	Reference	Reference	Reference	Reference	Reference	Reference
	4- < 6 h/day	0.65 (0.19–2.31)	0.69 (0.19–2.46)	0.72 (0.34–1.55)	0.71 (0.33–1.54)	1.11 (0.60–2.06)	1.13 (0.60–2.10)
	6–8 h/day	1.68 (0.63–4.50)	1.43 (0.50–4.10)	1.51 (0.80–2.83)	1.35 (0.71–2.58)	0.98 (0.54–1.79)	0.92 (0.50–1.69)
	> 8 h/day	0.92 (0.29–2.90)	0.88 (0.29–2.66)	0.90 (0.39–2.07)	0.76 (0.32–1.83)	1.22 (0.64–2.32)	1.12 (0.58–2.16)
Sleep duration							
	≥ 6 h/day	Reference	Reference	Reference	Reference	Reference	Reference
	< 6 h/day	0.80 (0.34–1.84)	0.83 (0.33–2.05)	1.02 (0.56–1.85)	1.00 (0.55–1.82)	0.93 (0.57–1.54)	0.99 (0.60–1.63)

^*^Adjusted for age, sex, race/ethnicity, and body mass index

## Discussion

Our study found that light physical activity level, sitting time, or sleep duration were not independently associated with a higher risk of progression to HCC in patients with cirrhosis. This lack of association remained when delineating among the different etiologies of cirrhosis, in different age groups, between males and females, with smoking status, or diabetes history. Furthermore, the composite effect of the three lifestyle variables showed no association with risk of progression to HCC when the groups with the highest and lowest levels of exposure for all three variables were compared.

The three variables of physical activity levels, sleeping time, and sitting time were selected because these potentially modifiable traits play a significant role in affecting all-cause mortality, cancer pathogenesis, and cancer survival [30–32]. For instance, a meta-analysis by Ekelund et al. found that individuals with the most amount of sitting time as a whole did not have increased mortality risk, but when narrowed to those who also had the least amount of physical activity, had significantly increased risk of death during follow-up. Hermelink concluded in another meta-analysis that high levels of sedentary behavior increased the relative risk of multiple types of cancer. Furthermore, existing data have shown that metabolic health and physical activity were associated with liver disease and that high BMI and poor physical condition, among other variables, had increased odds of MASLD development [22]. Moreover, mainstays of treatment for MASLD include weight loss, reducing sedentariness, and increasing exercise, indicating that physical activity and reduced sedentariness likely play a role in the development and pathogenesis of MASLD [33, 34].

For physical activity levels and sitting time, participants were relatively normally distributed between high and low exposure groups. Sleeping time was the only variable with an uneven distribution of participants across each variable’s subgroups, with 70% of participants sleeping more than 6 h per night. Even with this uneven distribution, no clear or significant associations arise. Etiology of cirrhosis was also considered in the analysis, as it is possible that metabolic causes of cirrhosis could be more likely to progress to HCC in patients whose metabolic health is poorer, versus cirrhosis caused by alcohol use or HBV/HCV. However, this was not the case, as demonstrated by the fact that there was no significant association across all etiologies of cirrhosis.

This is the first study investigating the association between physical activity levels, sleep, and sedentariness with the risk of progression of cirrhosis to HCC. One similar study, a prospective cohort study of 63,000 Chinese individuals aged 45 to 74, found a significant difference between the highest and lowest scores of a composite lifestyle score including BMI, smoking, alcohol use, diet, and sleep with a decrease in the risk of HCC development (HR: 0.13, 95% CI [0.06 – 0.30) [35]. Notably, this study did not account for the presence or development of cirrhosis in this cohort during the study period. Therefore, it is possible that the reported effect is a result of reduced risk of cirrhosis, rather than the risk of progression to HCC in patients already with cirrhosis and with sedentary lifestyles. Other studies assessing the link between cirrhosis and HCC have included obesity and other metabolic measures as risk factors, but not activity or sedentariness levels. For instance, one study by Nderitu et al. in a Swedish cohort shows that individual metabolic syndrome components, such as increased blood sugar, triglyceride levels, lipids, and obesity, are associated with increased risk of cirrhosis and primary liver cancer [9]. Another study by Xie et al. finds that poor physical condition and obesity traits significantly increase the risk of MASLD, a known risk factor for cirrhosis and HCC [36].

Based on our analysis, it could be the case that once cirrhosis is present in a patient, physical activity levels, sedentariness, and sleep duration do not affect HCC risk. However, it would be premature to consider this conclusion as definitive. Further investigation is necessary, especially among diverse patient populations in multiple geographic regions. Our use of survey data and necessity of using light activity as a measure represent measurement limitations, whereas data from heart rate monitors and sleep trackers would provide more objective measures. It is also worth noting that the time course captured in our study would not fully capture the effect of modifiable lifestyle factors on the development of HCC in patients with cirrhosis.

Our study examines the largest multicenter cirrhosis cohort study in the United States that includes patients of all ages, diverse racial and ethnic groups, and both sexes with cirrhosis from various etiologies, with minimal loss to follow-up. However, there are several limitations to this study. Even though steps were taken during recruitment and patient chart review to limit incorrect data gathering and loss of follow-up at all nine centers in this study, there is the possibility that patients who progressed to HCC were missed or that patients sought care at institutions outside of the THCCC. Misclassification of the self-reported physical activity levels in the last year, sleep duration, and sitting time in the past seven days was likely, but there is no clear reason for such errors to differ by eventual HCC status. Physical activity levels and sedentariness may also be affected by recall bias, social desirability bias, as well as cognitive limitations which may be present among cirrhosis patients who can manifest hepatic encephalopathy overtly or covertly. To address this issue, future studies should take advantage of technology available for objectively measuring sedentary time or physical activity as well as sleep duration and quality. The survey data limited physical activity level to only light physical activity without stratifying by intensity or type of activity. The survey also did not capture participants' sleep quality, light exposure at night, or other objective measures of sleep quality, sedentariness, or physical activity.

In conclusion, this is the first study investigating the potential impact of physical activity levels, sleep quantity, and sitting time on the progression of cirrhosis to HCC. We found that the risk of HCC development in patients with cirrhosis in our cohort was not significantly affected by level of physical activity or sleep quantity across all etiologies of cirrhosis and when examined jointly. Thus, even without a clear association between low levels of physical activity and the progression of cirrhosis to HCC, it is still important to consider the role of overall metabolic health in the progression of cirrhosis to HCC given the well-established impact metabolic health has on other aspects of health and chronic disease.

## Supplementary Information

Below is the link to the electronic supplementary material.

Supplementary file1 (XLSX 29 KB)

## Data Availability

Data may be available to other researchers with IRB approved protocols by contacting the corresponding authors (APT).
